# Intake of Artificial Sweeteners through Soft Drinks in the Preschool- and School-Aged Population

**DOI:** 10.3390/nu16142278

**Published:** 2024-07-15

**Authors:** Branka Jurcevic Zidar, Sanja Luetic, Katarina Jurcic, Zlatka Knezovic, Davorka Sutlovic

**Affiliations:** 1 Teaching Institute for Public Health, Split-Dalmatia County, 21000 Split, Croatia; branka.jurcevic.zidar@nzjz-split.hr (B.J.Z.); sanja.luetic@nzjz-split.hr (S.L.); katarina.jurcic@nzjz-split.hr (K.J.); 2 Department of Health Studies, University of Split, 21000 Split, Croatia; dsutlovic@ozs.unist.hr; 3 Department of Toxicology and Pharmacogenetics, School of Medicine, University of Split, 21000 Split, Croatia

**Keywords:** artificial sweeteners, soft drinks, children, consummation, labeling

## Abstract

One of the main public health issues that has recently been observed in a greater number of children is being overweight. The cause certainly lies in the decreasing physical activity of children, but mostly in their eating habits. Soft drinks are recognized as the most significant contributor to body overweight due to high sugar content; thus, as a result of numerous campaigns, part of the sugar is replaced by artificial sweeteners (ASs). Despite their advantage due to their low caloric value, WHO recommends that they should not be used to achieve weight control or as prevention for reducing the risk of non-communicable diseases, as there is no evidence of their effectiveness. Apart from beverages, artificial sweetener combinations are also added to a variety of “low fat” and “high protein” food products, which are highly favored especially among the young population. Therefore, it is necessary to take care of the cumulative intake. The conducted study included a survey of 323 parents of children aged 1–14 years, as well as an analysis of the AS content in the products most often consumed by the respondents. The results of the survey show that a large part of children (40%) aged 3–14 often consume soft drinks. Different products (soft drinks, juices/nectars, syrups) were sampled based on the respondents’ responses, and an analysis showed that 54% of them contained one or more ASs. In addition, the survey indicated parents’ lack of information about the presence of AS in products, as 51% of parents declared that they do not read the declarations of the products they buy. It is necessary to persist in consumer education and changes in dietary preferences and habits, especially among children.

## 1. Introduction

Among the most significant public health problems of modern society is the growing number of overweight and obese people. According to available data, as many as 53% of adults in Europe have a body mass index (BMI) above 25, while 29% of children aged 6–9 are overweight [1,2]. Excessive weight has been linked to the development of chronic non-communicable diseases, which are the primary cause of death, according to the findings of studies [3].

Excess body weight is most often the result of an improper diet followed by reduced physical activity. Despite numerous recommendations on creating healthy menus, the vast majority of residents of all ages consume significant amounts of sugar, which significantly affects their nutritional status. The World Health Organization (WHO) projected that 37 million children under the age of five would be overweight in 2022, which represents an increase of 4 million children worldwide compared to the year 2000. Excess body weight in early life is an additional risk for the development of chronic non-communicable diseases later in life [4]. Based on scientific evidence, the WHO in its guidelines recommends reducing the intake of free sugars in the total daily intake of calories to less than 10% in children and adults in order to reduce the risk of obesity and the development of dental caries [4].

In addition to the physiological role, food also has a hedonistic role, and the reduced sugar content often impairs the sensory properties of some types of food. Consumers, as well as food manufacturers, compensate for the diminished sweetness perception by adding artificial sweeteners. Sweeteners are food additives of different chemical composition and intensity of sweetness, and they are used to give food a sweet taste. Seventeen sweeteners and their combinations are currently approved in the European Union [5]. The most common sweeteners are artificial sweeteners, often called non-caloric (NC) and “natural” low-calorie sweeteners (LC), among which are polyols and steviol glycosides. The characteristics and comparison with natural sugars are shown in Table 1 [6,7,8].

The artificial sweeteners that are most often used in food production, individually or in combination are as follows: acesulfame potassium (acesulfame K), aspartame, cyclamate, neotame, saccharin and sucralose [9]. They are used in the production of a very wide range of food products from cereals, light dairy products, yogurts, desserts, bakery products, ice cream, puddings, cookies and candies with reduced sugar content, jams, syrups, canned fruits, chewing gums and low-calorie soft drinks.

The results of research from the United States of America NHANES (The National Health and Nutrition Examination Survey) from 2012 showed a consumption of artificial sweeteners of 41.4% in adults and 25.1% in children, which represented a significant increase compared to the year 1999 of 54% in the adult population and even 200% in children. Most of the sweeteners are introduced into the body by consuming low-calorie soft drinks (30.8% in adults and 19% in children) [10]. Studies conducted in several European countries also gave similar results, with children showing the greatest changes in their diet and consumption of non-alcoholic beverages. The results of these changes have undoubtedly led to disturbances in body weight and an increase in the number of children with an increased and excessively high body mass index (BMI) [11,12,13,14,15].

The intensive use of artificial sweeteners began in the 1990s and has been followed by very contradictory discussions ever since. A great advantage of artificial sweeteners is their strong sweetening power with a significantly lower caloric value compared to sugar, so they are often recommended for obesity control and consequently the prevention of hypertension, metabolic syndrome and caries prevention. However, the WHO recommendation is that artificial sweeteners should not be used as a means to reduce body weight and to reduce the risk of chronic non-communicable diseases [16]. Most artificial sweeteners are considered to have no harmful effects since they are not metabolized in the human body after absorption or are broken down into natural residual components [7]. However, in the case of high consumption, several studies point to their possible harmful effects in increasing the risk of type 2 diabetes [17], cardiovascular diseases, premature mortality [18,19] and changes in the gastrointestinal tract [9,20,21,22,23]. On the other hand, part of the studies does not link the aforementioned effects to the consumption of artificial sweeteners, indicating the need for further research in order to fully understand the effects of AS consumption on metabolic diseases [24,25,26].

In addition to promotional campaigns to reduce the amount of sugar in food, in some countries it is recommended to establish a system of taxation of sugar-sweetened soft drinks, the frequent consumption of which has been identified as a risk factor for the development of overweight [27]. In this way, manufacturers are encouraged to reduce the amount of sugar in their products.

European legislation prescribes the content and appearance of the label through which the consumer is given the necessary information about the product, its composition and the possible effects of some ingredients [28]. The regulation stipulates that the presence of sweeteners must be stated with the name of the food itself, which is usually not the case, but they are stated on the back, less visible part of the label. In addition to this practice of dishonestly informing consumers, according to the results of market research, a large number of average consumers who do not have any health problems usually do not read the declarations and are therefore completely unaware of what they are consuming in their food [29,30,31].

The results of the latest research conducted in the world, as well as in the Republic of Croatia, indicate a constant increase in overweight children. It is surprising that in Croatia the highest percentage of such children was recorded in coastal areas where nutrition is traditionally based on the principles of a healthy Mediterranean diet [32]. Soft drinks have been identified as one of the main causes of obesity problems. Therefore, the goal of this research was to determine whether and to what extent children of preschool and school age consume them. Also, it is planned to analyze different types of beverages that are shown in the survey to be frequently consumed for the presence of artificial sweeteners. It is also necessary to examine the attitude and awareness of parents regarding the presence and influence of artificial sweeteners in non-alcoholic beverages consumed. According to our knowledge, this is the first research of this type to be conducted in the Republic of Croatia.

## 2. Materials and Methods

A cross-sectional survey was conducted in Split, Croatia, in two large preschool institutions which have an organized diet. The menus do not contain beverages with ASs. Parents of school children were randomly selected.

### 2.1. Survey

Survey was conducted during March and April 2024. For this study, a questionnaire was developed to assess the consumption and attitudes of parents and children about the consumption of juices, various beverages and chewing gum. The poll included parents of preschool children aged 1–6.9 years (more precisely 6 years and 11 months) and parents of primary school children aged 7–14 years, and it was entirely voluntarily and anonymous. Questions on children and parents’ morphological status (age, height, weight and sport activity) were also included. Participants were also asked if they read the declarations on food products and if they have an attitude towards artificial sweeteners. The questionnaire was created by the researcher and was based on a review of the literature on this topic.

#### Survey Design

The study was approved by the Ethics Committee of University Department of Health Studies, University of Split (Class: 029-03/24-18/01). The questionnaire consisted of 26 questions. Questions were divided into 4 categories: general questions, questions about child consumption, questions about parental consumption and questions about parents’ attitudes and opinions. Attitudes were evaluated using a 5-point Likert scale (from strong disagreement to strong agreement), yes/no questions and categorical questions (with one or more choices).

### 2.2. Samples

Samples, 90 in total, of different drinks and chewing gums were taken from the Croatian market. Products were selected which the parents, participants in the survey, declared that they buy most often, regardless of whether they are Croatian or of foreign production. According to the type, the samples are grouped into 6 groups: soft drinks (35 samples), fruit juice/nectars (33 samples), syrups (3 samples), instant drinks (5 samples), protein drinks (2 samples) and chewing gums (12 samples).

### 2.3. Determination of Acesulfame Potassium, Sodium Saccharin Dihydrate and Aspartame

The determination of acesulfame potassium, sodium saccharin dihydrate and aspartame has been carried out according to HRN EN 12856:2000 [33].

#### 2.3.1. Chemicals and Working Solutions

The acetonitrile and methanol, HPLC grade, as well as potassium dihydrogen phosphate, were purchased from Merck Co. (Darmstadt, Germany). The standards of acesulfame potassium and aspartame were bought from Sigma Aldrich (St. Louis, MO, USA), while sodium saccharin dihydrate was purchased from Merck Co. (Darmstadt, Germany).

Deionized water (0.05 mS cm^−1^) was prepared from the ultrapure water system Omnia Pure (Stakpure GmbH, Niederahr, Germany). Standard stock solutions of acesulfame potassium, sodium saccharin dihydrate and aspartame (1000 mg L^−1^) were prepared by dissolving in deionized water; working solutions were prepared by serial dilution of the stock solution.

#### 2.3.2. Sample Preparation

Carbonated samples were degassed before preparation (15 min at room temperature) in an ultrasonic bath Sonis 4GT (Iskra, Ljubljana, Slovenia). In total, 20 mL of the homogenized sample was diluted to a volume of 100 mL with deionized water and filtered through a 0.45 μm syringe filter (Labex Ltd., Budapest, Hungary). Prepared samples were immediately analyzed on HPLC.

#### 2.3.3. Method

The analysis was carried out on the Agilent 1200 HPLC system (Agilent Technologies Singapore (International) Pte. Ltd., Singapore) equipped with a diode array detector (190–400 nm) and OpenLab CDS Chemstation Software (Agilent, Singapore, rev. B.04.03) and the Agilent 1260 HPLC system (Agilent Technologies Singapore (International) Pte. Ltd., Singapore) equipped with a diode array detector (190–900 nm) and OpenLab CDS Chemstation Software (Agilent, Singapore, rev.C.01.05). For the separation, an Agilent Zorbax reversed-phase HPLC column (250 × 4.6 mm; 5 µm) was used with a flow of 1.0 mL min^−1^ and an injection volume of 20 μL. The mobile phase was 0.05 M solution of potassium dihydrogen phosphate (A) and acetonitrile (B); (90:10, *v*/*v*, gradient elution—0 min: 90% A:10% B/10 min: 60% A:40% B). Potassium dihydrogen phosphate (0.05 M) was prepared by dissolving in deionized water; the pH was adjusted to 3 with 5% phosphoric acid. The maximum wavelength was 210 nm for aspartame and sodium saccharin dihydrate and 226 nm for acesulfame potassium. 

The limit of quantitation (LOQ) was 2.5 mg L^−1^ for all three analytes. The mean recovery was ≥ 95.0% with a linearity (R^2^) of 0.999 for all analytes. All samples were analyzed in duplicate, with an RSD < 5%. The results are reported as the mean value.

### 2.4. Determination of Cyclamate

The determination of cyclamate has been carried out according to HRN EN 12857:1999 [34].

#### 2.4.1. Chemicals and Working Solutions

Methanol and n-heptane, HPLC grade, were purchased from Merck Co. (Darmstadt, Germany) and cyclamate was purchased from Sigma Aldrich (St. Louis, MO, USA).

All chemicals used for analyte extraction (potassium-hexacyanoferrate, zinc sulphate) and derivatization (sulfuric acid, sodium sulphate, sodium carbonate, potassium iodide, hydrochloric acid, 0.1 mol L^−1^ sodium thiosulphate and starch) were purchased from Merck Co. (Darmstadt, Germany), while sodium hypochlorite solution was purchased from Medimon (Split, Croatia).

#### 2.4.2. Sample Preparation

The liquid samples were diluted with deionized water to a cyclamate concentration of about 400 mg L^−1^. Carbonated beverages were degassed in an ultrasonic bath before analysis. Solid products (chewing gums) were dissolved in water. Dairy, semi-solid products were diluted in water, and Carrez I solution (15% aqueous solution of potassium hexacyanoferrate trihydrate) was added to eliminate the interfering compounds and turbidity. Samples were than filtered through filter paper.

The prepared samples were derivatized in a separating funnel using a mixture of sulfuric acid, n-heptane and sodium hypochlorite according to the procedure described in the standard HRN EN 12857:1999 [34]. The n-heptane layer was filtered through filter paper with anhydrous sodium sulphate. The derivatized samples were filtered through a 0.45 μm syringe filter (Labex Ltd., Budapest, Hungary) and immediately analyzed by HPLC.

#### 2.4.3. Method

The analysis was carried out on the Agilent 1200 HPLC system and Agilent 1260 HPLC system (details in Section 2.3.3). For the separation, a reversed-phase C18 column (250 × 4.0 mm; 5 μm) with a precolumn was used with a flow of 1.0 mL min^−1^ and an injection volume of 20 μL. The mobile phase was a mixture of 80% methanol and 20% deionized water. The maximum wavelength was 314 nm. 

The limit of quantitation (LOQ) was 2.5 mg L^−1^. The mean recovery was ≥95.0%, with a linearity (R^2^) of 0.999. All samples were analyzed in duplicate, with an RSD < 5%. The results are reported as the mean value.

### 2.5. Statistical Analysis

Data analysis utilized descriptive statistics to describe responses to survey items. The differences between the groups of study parameters were measured using the Chi-square and Mann–Whitney U tests. Differences and relationships were considered to be statistically significant at a *p*-value < 0.05. 

The Kolmogorov–Smirnov test was used for the analysis of the distribution of normality for AS concentrations. Statistical analysis was performed using Statistical Package Software for Social Science, version 26 (SPSS Inc., Chicago, IL, USA).

## 3. Results and Discussion

Data on the research participants (both parents and children), filled out by the parents, are displayed in Table 2. A total of 323 parents completed the survey: 250 for preschool children aged 1–6.9 and 73 for school children aged 7–14.

In the preschool group, ten children were aged under 2 years and, due to their young age, they were excluded from statistical processing and correlations with drink consumption and physical activities. Their parents answered that they do not consume any of the mentioned drinks and do not participate in any activities.

The majority of the parents who filled out the survey were women, 89% (287 out of 323 respondents). The median age of all respondents (parents) was 37 years.

Out of the total number of respondents, 145 parents answered that they eat more than three meals a day with their child. This indicates that children are mostly fed at home and parents develop their eating habits. A statistically significant difference between parents’ BMI and children’s BMI was observed by the Mann–Whitney U test (*p* < 0.05). The median value in the parents’ group was 23 and it was 15.4 in the children’s group. The BMI of most children (83%) was below the recommended range according to the WHO which is 18.5–24.9 [35]. Fifty-two children had a BMI within the recommended limits while only one child had a BMI above 24.9; more precisely, his BMI was 28.3. His parent had a BMI of 30, which is above the upper recommended limit. 

**Figure 1:**
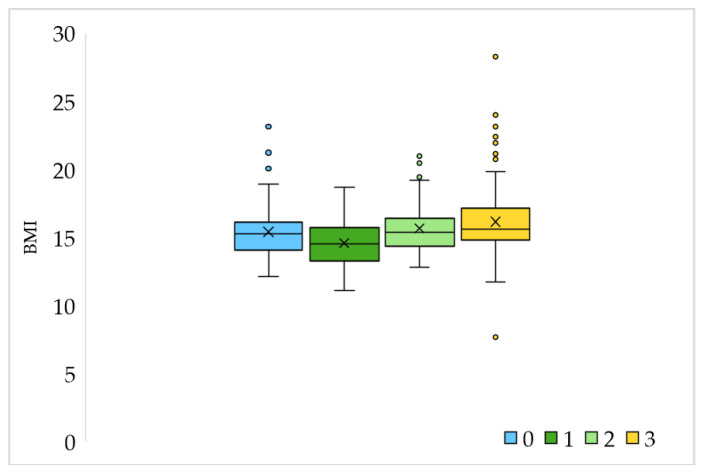
Correlation of BMI and children´s physical activity; 0 = does not exercise, 1 = exercise once a week; 2 = exercise twice a week; 3 = exercises 3 or more times a week.

Physical activity is important in maintaining health, so one of the questions in the survey is dedicated to that topic. Of the total number of children (313) aged 2–14, 104 of them (33.2%) do not engage in any sports activities. For the rest, the parents declared that they attend some form of physical activity once a week (4.2%), twice a week (26.8%) or three or more times (35.8%). The correlation between children’s BMI and physical activity is shown in Figure 1.

**Figure 2:**
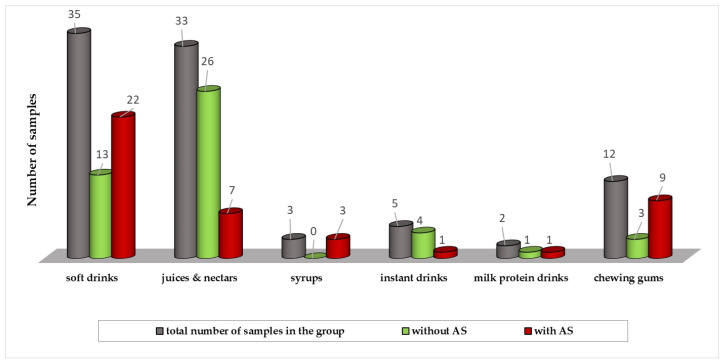
Distribution of the samples according to the AS content.

The results of the AS determination (acesulfame K, aspartame, cyclamate and saccharine) in samples of various beverages and chewing gums are shown in Figure 2.

A total of 78 beverage samples and 12 chewing gum samples were analyzed. Out of the total number of drinks, 42 samples (54%) contained the mentioned artificial sweeteners in different combinations. It should be noted that the brands of products that the parents said in the survey they purchase most frequently were the ones that were sampled and analyzed.

In the Republic of Croatia, the Law on the Special Tax on Sugar is in force, which aims to stimulate manufacturers to reduce the amount of sugar in soft drinks. Therefore, manufacturers compensate for the reduced taste of sweetness with ASs. Artificial sweeteners were found in 75% of the examined chewing gum samples, 59% of soft drinks and 21.2% of juices and nectars. In most samples, artificial sweeteners were declared in the composition of the product and proved by analysis. Only in one sample did the analysis prove the presence of ASs which were not mentioned on the label. 

Table 3 shows the results of the analysis for four types of artificial sweeteners in the sampled products. The Kolmogorov–Smirnov test was used for the analysis of the distribution of normality. Since the distribution of our results did not follow a normal distribution, the median and concentration range are displayed.

Overall, 5 samples out of a total of 35 soft drinks were declared “zero sugar”, which is the generally recognized name for sugar-free products that contain ASs. In the case of the other 22 samples, the product name did not state that they were soft drinks with sweeteners, so the average consumer, who does not read the labels carefully, may not be aware that the product contains ASs. The results of the questionnaire confirm exactly that, since 50.8% of parents answered that they do not read or only sometimes read the product labels. In the group of juices and nectars, AS was found in seven samples, where only one of these samples was declared as “zero sugar”. The other samples had AS listed in the composition, but not in the product name. Moreover, in one sample in which ASs were not labeled, two of them were found. From the results of the survey, it was possible to conclude that most parents do not distinguish between two groups of drinks and that they actually buy nectars thinking they are buying original fruit juices. The difference between juice and nectar is in the fruit content. Juice has 100% fruit content with no added sugar, while nectar is a drink made from fruit juice or pulp, water and sugar. Most often, product packaging with large pictures of fruit suggests that it is fruit juice. Not a single nectar sample in our study did not contain statement with AS in the name of the product, which is mandatory according to legal regulations [28].

**Figure 3:**
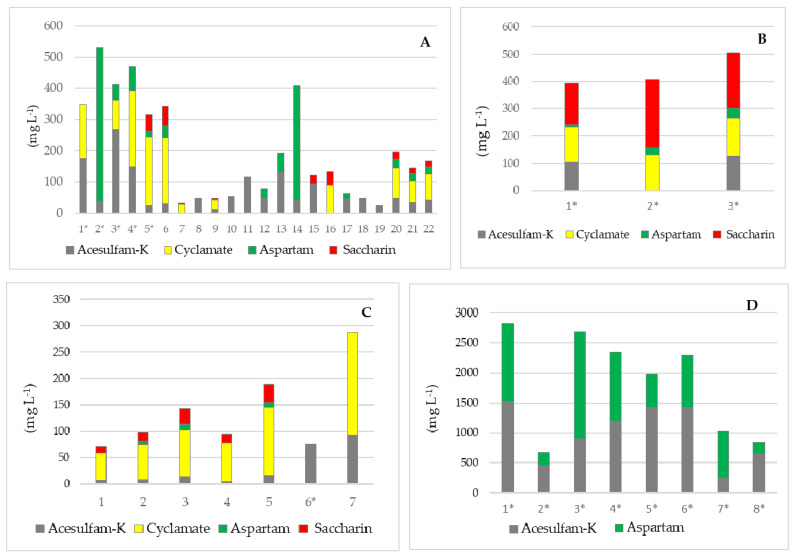
Distribution of different ASs in samples analyzed within this study; numbers on the X-axis present the serial number of the sample (* samples declared as zero sugar); (**A**)—samples of soft drinks; (**B**)—samples of syrups; (**C**)—samples juices and nectars; (**D**)—samples of chewing gums; the Y-axis represents the total concentration of all artificial sweeteners in each sample.

Most of the samples in which ASs were found contained several types of sweeteners, with acesulfame K and aspartame being the most represented, in 93% and 68% of the samples, respectively. The combination of several types of sweeteners is common in recipes because in this way a greater feeling of sweetness is achieved, and there is no danger of exceeding the individual maximum concentrations prescribed by Regulation 1333/2008. In some samples, the concentration of individual AS was very slightly below the prescribed limit concentration, along with the fact that the sample also contained other sweeteners. By combining several artificial sweeteners, the optimal sweet taste of the product is achieved, because the undesirable characteristics (bitter or sour taste) that some sweeteners have are masked [36]. Figure 3 shows the results of analysis for products that contained several types of AS.

Acesulfame K was found in 91% of the 32 beverage samples where artificial sweeteners were detected. Although acesulfame K is considered to have no harmful effects on health, recent research indicates a possible connection between the consumption of acesulfame K and the occurrence of atherosclerosis and glucose intolerance [24,37].

**Figure 4:**
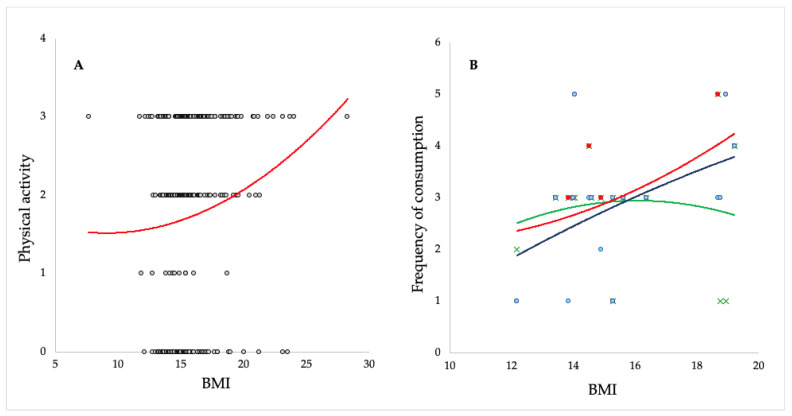
(**A**)—correlation between the BMI and physical activity of the children involved in this study (the number on the Y-axis indicates the number of weekly physical activity); (**B**)—correlation between consumption of soft drinks and syrups and BMI of children in this study (the number on the Y-axis indicates the frequency of consummation, from none to several times a week. Trendline curves shown in different colors: green for juice/nectars, blue for soft drinks and red for syrups).

Aspartame was found to be the next most represented sweetener in 56% of the beverage samples analyzed. Most artificial sweeteners, including aspartame, are considered to have no harmful effects since they are not metabolized in the human body after absorption or are broken down into natural residual components [7]. Since aspartame has poor stability at various pH and temperatures, its decomposition usually produces aspartic acid (ASP), phenylalanine (PHE), aspartyl-phenylalanine (ASP-PHE) and 5-benzyl-3,6-dioxo-2-piperazieacetic acid (DKP) [38]. According to EFSA scientific opinion on the safety of aspartame, there is no safety concern with the exposure of 40 mg/kg b.w./day [39]. Still, some recent studies indicate that in beverages stored in transparent bottles, aspartame breakdown products can arise as a result of sunlight exposure [40]. The research revealed phenolic derivatives that have been identified for the first time and which according to the first in silico predictions can have potentially harmful effects [40]. The results of the survey showed that 39.0% of parents regularly purchase and consume soft drinks and similar beverages, and 36.4% stated that their children also consume this type of beverage more than once a week, some even several times a day. Children aged 2 to 14 years (18.8%) also regularly consume syrups for dilution in which different combinations of several types of AS have been proven. ASs have no caloric value, so their frequent consumption can cause an increased need for food and an energy imbalance between intake and energy consumption. Children with reduced physical activity are especially susceptible to weight gain. Analysis of the results of the survey reveals a disparity between the BMI and physical activity of children aged 3 to 14 years. The results are shown in Figure 4A, where it is possible to observe a visible trend of increasing BMI despite greater sports activity, which may be a consequence of improper nutrition. Although BMI has certain limitations because it does not provide information on body composition (subcutaneous fat tissue and muscle mass), it is still most often used as an indicator of the level of nutrition [41,42].

Through data analysis, we tried to find out whether the consumption of juice, soft drinks and syrup for dilution was correlated with BMI for a group of children aged 3 to 7 whose parents indicated in the survey that they frequently drink all three types of drinks. Figure 4B shows a positive correlation between the frequent consumption of soft drinks and syrups and the increase in BMI for the aforementioned group of children. In addition to the high sugar content, all these drinks contained a combination of several ASs. According to some studies, the frequent occurrence of AS indicates possible changes in metabolic pathways that are relevant for appetite control and energy balance [43].

Chewing gum contains AS in large concentrations and usually more than one AS. The results of our survey showed that respondents aged 3 to 14 did not consume chewing gum to a significant extent. Out of the total number of respondents, only eight of them consumed gum once a day, while only one respondent consumed five gums per day.

The results of our research are in accordance with research conducted in Germany where the presence of aspartame in different types of food and drinks has been investigated [44]. The authors highlighted the possible issue of consuming aspartame cumulatively through a variety of food sources. Therefore, in our research, in addition to refreshing drinks and syrups, we also included various “low fat” and “high protein” dairy products that are very popular among the young population. The analysis of the sweetener was carried out on only two samples, one of which did not contain AS while the other contained acesulfame K. However, a larger number of product declarations on the market were reviewed. This type of product is accompanied by strong marketing, highlighting the high proportion of protein and the beneficial effect on the body. After reviewing the labels, it was observed that the product name did not contain any indication of the presence of sweeteners, and most of them had two ASs in the declared composition.

Our research included children aged 1 to 14 years, where proper nutrition is extremely important for growth and development. Numerous studies in recent years have indicated the importance of the intestinal microbiome and its key role in the regulation of metabolism, the body’s immune response and the preservation of overall human health. A varied and high-quality diet is an important factor that affects the development of the intestinal microbiome. Basson et al. discuss the potential impact of AS on the intestinal microbiome, the intestinal wall’s immune response and the detrimental consequences on the progression of chronic inflammatory bowel disease [23]. Artificial sweeteners can disrupt the balance of microbiota by encouraging the growth of certain bacteria and suppressing others. According to some studies, an excessive consumption of artificial sweeteners can change the expression of genes involved in carbohydrate metabolism, disrupting the growth and balance of intestinal bacteria, which can lead to metabolic and inflammatory changes that can cause the development of cardiovascular diseases [18,19]. The results of some recent studies suggest that the most commonly consumed ASs (aspartame, acesulfame K, sucralose and saccharin) can affect the gut microbiome and potentially raise antibiotic resistance by altering bacterial cell permeability and cell membranes [45,46]. It is possible that aspartame and its metabolites cause alterations in the gut microbiota and glucose intolerance [21,22], as well as the development of type 2 diabetes [47].

On the other hand, different researchers have shown that there is no clear evidence of AS consumption on body weight and glycemic control [48], as well as their adverse impact on the gut microbiota if they are consumed at the recommended levels [49,50]. Also, some studies suggest that there are no strong arguments confirming the connection between non-nutritive sweeteners’ consumption and an increased risk for diabetes [51]. Conflicting findings from preclinical and clinical research have been reported due to a lack of appropriate biomarkers indicating the need for continued investigation in order to determine the actual effects of ASs. In the literature, a high consumption of aspartame is associated as a trigger for headaches and migraines in people who are prone to these diseases, and it is also associated with the appearance of dizziness, changes in mood, irritability and changes in spatial orientation.

Until all doubts are clarified with certainty, it is recommended for pregnant women to avoid aspartame intake during pregnancy and breastfeeding, as well as people with phenylketonuria for which aspartame is completely prohibited [52]. An additional problem is that the quantities of added additives are not listed on the declarations, so it is not possible to estimate the daily intake per person. Also, in the majority of analyzed samples in which sweeteners were present, several types of AS were proven, which increased the cumulative intake.

### Study Limitations

This study had several limitations. The questionnaire used was not validated and we do not have accurate data on the amount and type of all food containing ASs consumed by children. It is necessary to validate this questionnaire and identify all types of foods and portions consumed daily in order to more accurately determine the amount of ASs consumed by children. One limitation of the study is its reliance on the answers of parents who may have answered some questions under the pressure of socially acceptable attitudes. In order to obtain more information about the intake of artificial sweeteners, it is necessary to analyze a larger number of products, in particular samples of low-fat and high-protein milk drinks in order to determine which types of ASs and what concentrations they contain. 

## 4. Conclusions

The results of our research confirm that a large part of the beverages on the Croatian market (soft drinks, juices and nectars, as well as syrups) contain artificial sweeteners. According to the results of the survey, a large number of children (40%) regularly consume various beverages containing ASs. Since there is an increasing number of different food products with ASs on the market, it is necessary to take care of the cumulative intake.

Taking into account the increasingly widespread use of ASs, it is important to determine to what extent and from which sources the young population consumes artificial sweeteners and whether these values cross over acceptable daily intakes. 

It is necessary to persist in consumer education, because the fact that about 50% of the surveyed parents were uninformed about the characteristics of the food they give their children is a warning. 

Based on the presented results, it is clear that it is necessary to plan and implement education campaigns for parents and children in order to change eating habits. It is very important, especially for young children, to change the perception of food acceptance and become used to the consumption of normal, not overly sweetened food, whether the sweetness comes from sugar or artificial sweeteners.

## Figures and Tables

**Table 1:** Characteristics of different sweeteners, natural and artificial, degree of sweetness, caloric value and acceptable daily intake (ADI).

Sweeteners	* Degree of Sweetness	kcal g^−1^	ADI mg kg^−1^ b.w.	Natural Sugars	* Degree of Sweetness	kcal g^−1^	ADI mg kg ^−1^ b.w.
**Artificial sweeteners**							
Advantame	37,000	0	5	Fructose	1.1–1.5	4	not specified
Aspartame	200	4	40	Sucrose	1	4	not specified
Acesulfame K	150–200	0	9	Dextrose	0.9	4	not specified
Cyclamate	30–80	0	11	Glucose	0.75	4	not specified
Neotame	7000–13,000	0	2	Maltose	0.4	4	not specified
Neohespedrin DC	1500–2000	2	20	Galactose	0.3	4	not specified
Sucralose	400–800	0	15	Raffinose	0.2	4	not specified
Saccharin	240–300	3.6	5				
**Natural sweeteners**							
***Natural plant protein***							
Thaumatin	3000	4.0	not specified				
***Sugar alcohols–polyols***							
Lactitol	0.35	2	not specified				
Xylitol	1	2.4	not specified				
Erythritol	0.7	0.2	0.5				
Maltitol	0.75	2.1	not specified				
Mannitol	0.6	1.6	not specified				
Sorbitol	0.6	2.6	not specified				
Isomaltose	0.55	2	not specified				
Steviol glycosides	250	0	4				

* Comparison of the degree of sweetness on the assumption that the sweetness equivalent of sucrose = 1.

**Table 2:** Characteristics of participants (N = 323).

Variable		Age 1–6.9 N = 250	Age 7–14 N = 73	Difference between Groups (According to Age) χ2 Test
Gender	F	108	37	*p* = 0.258
	M	142	36	
Age	Minimum	1.0	7.0	
	Maximum	6.5	14.0	
	Average ± S.D.	4.42 ± 1.44	8.92 ± 2.0	
BMI	Minimum	11.11	7.65	
	Maximum	28.28	24.0	
	Average ± S.D.	15.49 ± 1.99	16.4 ± 2.69	
BMI Parents *	Minimum	16.61	19.15	
	Maximum	36.82	36.01	
	Average ± S.D.	23.13 ± 3.21	24.59 ± 4.20	
Sports activities	Not	100	14	*p* < 0.05
	Yes, 1 time a week	11	2	
	Yes, 2 times a week	67	17	
	Yes, 3 or more times a week	72	40	
Number of common meals	0	0	2	*p* = 0.113
	1	30	10	
	2	108	28	
	3	60	19	
	More than 3	52	14	

(*) data for parents.

**Table 3:** Distribution and levels of ASs (mg kg^−1^) in the different types of drinks and chewing gums in this research.

	Sample	Soft Drinks	Juices/Nectars	Syrups	Instant Drinks	Milk Protein Drinks	Chewing Gums
	No. of samples	35	33	3	5	2	12
Acesulfame K	Med (mg L^−1^)	18.1	<LOQ	116.7	<LOQ	<LOQ	557.2
	Conc. range (mg L^−1^)	<LOQ–268.7	<LOQ–92.5	106.0–127.4	<LOQ–172.7	<LOQ–122.6	<LOQ–1520.4
Cyclamate	Med (mg L^−1^)	<LOQ	<LOQ	131.3	<LOQ	<LOQ	<LOQ
	Conc. range (mg L^−1^)	<LOQ–241.0	<LOQ–195.0	125.6–138.2	-	-	-
Aspartame	Med (mg L^−1^)	<LOQ	<LOQ	25.2	<LOQ	<LOQ	558.3
	Conc. range (mg L^−1^)	<LOQ–493.0	<LOQ–10.2	10.4–37.1	-	-	<LOQ–1791.5
Saccharin	Med (mg kg^−1^)	<LOQ	<LOQ	202.1	<LOQ	<LOQ	<LOQ
	Conc. range (mg kg^−1^)	<LOQ–63.1	<LOQ–33.9	150.5–250.4	<LOQ–622.7	<LOQ	-

Med = median; LOQ (limit of quantitation) = 2.5 mg L^−1^.

## Data Availability

The data presented in this study are available upon request from the corresponding author.
